# Relationship between *PIWIL1* gene polymorphisms and epithelial ovarian cancer susceptibility among southern Chinese woman: a three-center case–control study

**DOI:** 10.1186/s12885-023-11651-2

**Published:** 2023-11-27

**Authors:** Shanshan Liu, Yaping Yan, Zhizhong Cui, Haipeng Feng, Fengmei Zhong, Ziguang Liu, Yan Li, Xiang Ou, Wenjuan Li

**Affiliations:** 1 Department of Hematology and Oncology, Guangzhou Women and Children’s Medical Center, Guangzhou Medical University, Guangdong Provincial Clinical Research Center for Child Health, Guangzhou, 510623 China; 2 Department of Traditional Chinese Medicine, The Third Affiliated Hospital of Guangzhou Medical University, Guangzhou, 510150 Guangdong Province China; 3 Department of Pathology, Shunde Hospital, Southern Medical University, Foshan, 528000 Guangdong China; 4 Medical Research Center, Shunde Hospital, Southern Medical University, Foshan, 528000 Guangdong China; 5 The Affiliated Changsha Central Hospital, Hengyang Medical School, University of South China, Changsha, China

**Keywords:** Epithelial ovarian cancer, *PIWIL1*, PiRNA, Susceptibility, Polymorphism

## Abstract

**Objective:**

To investigate the potential correlation between piwi-like RNA-mediated gene silencing 1 (*PIWIL1*) polymorphisms and susceptibility to epithelial ovarian cancer (EOC).

**Methods:**

A case–control study was conducted to evaluate the susceptibility of EOC using multinomial logistic regression analysis. The study analyzed the relationship between five functional single nucleotide polymorphisms (SNPs) in the *PIWIL1* gene and EOC risk. Genotyping of 288 cases and 361 healthy samples from South China was identified using a TaqMan assay. Odds ratios (ORs) and 95% confidence intervals (CIs) were calculated to estimate the relationship between the five selected SNPs and EOC susceptibility.

**Results:**

Among the five SNPs analyzed, the rs10848087 G > A and rs7957349 G > C variants significantly increased the susceptibility of EOC, rs10773771 C > T was associated with a decreased risk of EOC, while the rs35997018 and rs1106042 variants were not in Hardy–Weinberg equilibrium (*p* < 0.05). The rs10848087 G > A was significantly associated with increased risk of EOC in individuals with metastasis, FIGO stage I and III, low and high pathological grade, tumor numbers ≤ 3 and > 3, tumor size > 3 cm and ≤ 3 cm, pregnant more than 3 times, pre-menopausal status, and strong positive expression of ER (estrogen receptor), PR (progesterone receptor), PAX8 (paired-box 8), wild-type p53 (tumor protein 53), WT1 (Wilm’s tumor gene), P16 (cyclin-dependent kinase inhibitor 2A). In addition, rs10848087 G > A enhanced the EOC risk of cases with negative/mild positive expression of wild p53 and Ki67, and with or without mutant p53 expression. The rs7957349 G > C variant was linked to an increased risk of EOC in subgroups with certain characteristics, including age equal or less than 53 years, metastasis, clinical stage I, low pathological grade, tumor number, tumor size, pregnant times, post-menopause, pre-menopause, and strong positive expression of wild p53 and Ki67 (Antigen identified by monoclonal antibody Ki-67), as well as without mutant p53 expression. The rs10773771 CT/TT alleles were identified to have a protective effect on EOC in women aged 53 years or older, as well as in cases with metastasis, advanced clinical stage, high pathological grade, multiple tumors, tumor size equal to or less than 3 cm, history of pregnancy, post-menopausal status, and strong positive expression of ER, PR, wild-type p53, PAX8, WT1, P16, and Ki67. Furthermore, rs10773771 CT/TT also showed a protective effect in patients with negative or mildly positive expression of PR, PAX8, wild-type p53, WT1, and P16, as well as positive expression of mutant p53. Compared to the reference haplotype GCG, individuals harboring haplotypes GTG were found to have a significantly decreased susceptibility to EOC. PIWIL1 was significantly expressed in the thyroid, pituitary, and adrenal glands with rs7957349 CC alleles.

**Conclusions:**

*PIWIL1* rs10848087 and rs7957349 were associated with increased risk of EOC, while rs10773771 may have a protective effect against EOC. These genetic variants may serve as potential biomarkers for EOC susceptibility in the South China population.

**Supplementary Information:**

The online version contains supplementary material available at 10.1186/s12885-023-11651-2.

## Introduction

Ovarian cancer is the second most common cause of gynecologic cancer death in women worldwide [1]. There are three major types of ovarian cancer-epithelial, germ cell, and sex cord-stromal tumors. Epithelial ovarian cancer (EOC) is a highly heterogeneous phenotype with five major histotypes for invasive disease-high-grade serous, low-grade serous, endometrioid, clear cell, and mucinous histotype [2]. It accounts for approximately 3.4% and 4.7% of new cancer cases and deaths among women worldwide [3]. Unfortunately, due to the lack of specific clinical manifestations and mature screening techniques in the early stage, most EOC cases are already diagnosed in the late stage (advanced III-IV), and cancer has already spread and metastasized in the abdominal cavity [4]. As a result, approximately 70% of cases have reached the late stage without early warning symptoms [5]. The 5-year survival rate for this disease is around 45% [6], which is relatively low due to poor pathological features resulting in limited clinical efficacy [7].

Early diagnosis is an optimal strategy for enhancing the dismal survival rates associated with ovarian cancer. If the disease is detected at early stages (IA and IB), characterized by small or localized tumors, approximately 93% of patients can achieve a five-year survival rate after diagnosis [8, 9]. Diagnostic testing for symptomatic individuals includes physical examination and radiological imaging techniques such as transvaginal ultrasound (TVUS). However, there are currently no available screening strategies for asymptomatic women to detect ovarian cancer in its early stages. The most promising screening tools presently include the cancer antigen 125 (CA125) blood test and TVUS [10, 11]. Additionally, human epididymis protein 4 (HE4) has been investigated as a potential biomarker for ovarian cancer screening; however, further studies are warranted [10]. The medical and surgical treatment strategies for epithelial ovarian cancer in women are continuously evolving. In recent years, significant progress has been achieved, supported by groundbreaking clinical trials. Although the treatment of primary epithelial ovarian cancer still involves a combination of surgery and systemic therapy, there is now a standardization of more intricate surgical procedures and novel therapeutic approaches [12, 13]. Cytotoxic chemotherapy and maximal surgical efforts remain the mainstream approach; however, targeted therapies are increasingly being utilized as well, while new data have raised questions regarding the role of surgery in women with recurrent disease [14, 15]. Poly-ADP-ribose polymerase inhibitors have demonstrated improved progression-free survival rates in both first-line and recurrent patients, leading to their increasing utilization [16]. The recent classification based on genetic alterations further emphasizes the recommendation for germline genetic testing in all women diagnosed with epithelial ovarian cancer [17–20], while new drug approvals driven by biomarker analysis suggest potential benefits from somatic molecular testing as well.

*PIWIL1*, piwi-like RNA-mediated gene silencing 1, a member of the PIWI subfamily of Argonaute proteins, is involved in stem cell self-renewal, RNA silencing, and translational regulation in various organisms. *PIWIL1* may, as an oncogene, be overexpressed in multiple types of tumors, such as gastric cancer [21], lung cancer [22], hepatocellular carcinoma [4], pancreatic adenocarcinoma [23], and endometrial cancer [24]. *PIWIL1* Knocking out the *PIWIL1* gene (PIWIL1-KO) has significantly reduced gastric cancer cell proliferation, migration, metastasis, and tumorigenesis [21, 25]. In pancreatic ductal adenocarcinomas, Feng Li et al. [26] discovered that human *PIWIL1* functions as an oncoprotein, activating the anaphase-promoting complex/cyclosome E3 complex in the absence of piRNAs, this complex targets a critical cell adhesion-related protein to enhance PDAC metastasis. Cheng et al. [27] observed that the RASSF1C-PIWI-piRNA pathway promotes lung cancer cell growth and progression and suggests that *PIWIL1* protein is abnormally expressed in various types of cancer, making it a potential biomarker and therapeutic target. Wen et al*.* [28] reveal that *Piwil* serves as a crucial regulator gene in germ cell division during gonadal development and is closely associated with germ cell differentiation. A total of 219, 256, and 234 piRNAs were detected in normal ovary, endometrioid, and serous ovarian cancer samples, respectively. The functional analysis of the predicted targets of differentially expressed piRNAs revealed their potential to modulate key processes and pathways involved in ovarian oncogenesis [29]. However, it is important to note that these findings are just the tip of the iceberg, as the relationships between the piRNA pathway and ovarian cancer progression have not yet been extensively studied.

Additionally, allele-specific DNA methylation differences at regulatory sites of genes involved in piRNA regulation have been linked to impaired spermatogenesis [30]. While a single-nucleotide polymorphisms study (SNPs) has shown that the GG genotype and G allele of rs28416520 within the *PIWIL1* gene promoter CpG 67 region are associated with an increased risk of gastric cancer [31], the impact of SNPs of *PIWIL1* gene on EOC risk has not been studied.

The predominant histological subtypes of ovarian cancer encompass high-grade serous carcinoma, low-grade serous carcinoma, endometrioid carcinoma, clear cell carcinoma, and mucinous carcinoma. Nuclear antigens comprise cell cycle-associated proteins (such as P16 and Ki-67), tumor suppressor gene products (such as p53 and WT1), and steroid hormone receptors (such as ER and PR). Notably, the expression patterns of WT1, ER, PR, mutant P53, and wild-type P53 can effectively discriminate between different types of ovarian cancer [32, 33]. PAX8 can be used to distinguish primary ovarian cancer from metastatic cancer [34, 35]. Additionally, PAX8 can serve as a useful tool in distinguishing primary ovarian cancer from metastatic cancer [34, 35]. In the context of ovarian cancer prognosis analysis, increased expression of p16 and Ki67 has been associated with more aggressive tumor growth patterns and poorer clinical outcomes [36]. These aforementioned markers have been employed in our stratification analysis.

Given the evidence that the *PIWIL1* gene promoted tumorigenesis, we conducted a three-center case–control study to explore the association between genetic variations in the *PIWIL1* gene and the risk of EOC in southern Chinese women.

## Materials and methods

### Patients and healthy controls

A total of 288 EOC patients and 361 healthy controls, ranging from 20 to 88 years old, were recruited from Guangzhou Women and Children's Medical Center, Shunde Hospital of Southern Medical University, and The First Affiliated Hospital of Jinan University between 2016 and 2022. The diagnosis was confirmed by two pathologists independently, and tumors were classified according to the WHO Classification of Tumors of the female genital tract [37]. All participants in this study provided written informed consent, and the Ethics Committee of Guangzhou Women and Children's Medical Center (117A01) and Shunde Hospital of Southern Medical University (KYLS20220903) approved the research. The experiments were conducted by the Declaration of Helsinki. The demographic characteristics of all participants are presented in Table S1. The *PIWIL1* gene SNPs rs10848087 G > A, rs7957349 G > C, and rs10773771 C > T were stratified based on various factors including age, metastasis, clinical stage, pathological grade, tumor number, tumor size, pregnancy history, menopausal status, and expression levels of ER, PR, PAX8, wild-type p53, mutant p53, WT1, P16, and Ki67 (refer to Table 2 for details).

### SNP selection and genotyping

Five SNPs (rs10848087 G > A, rs35997018 T > C, rs10773771 C > T, rs7957349 G > C, and rs1106042 G > A) were selected from the NCBI dbSNP database (http://www.ncbi.nlm.nih.gov/projects/SNP) based on previously described criteria [38]. The potential functions of these SNPs were evaluated using the SNPinfo online server (http://snpinfo.niehs.nih.gov/snpfunc.htm). Genomic DNA from patient samples was extracted using the TIANamp DNA Kit (Tiangen, Beijing, China) from paraffin-embedded tissue. In contrast, genomic DNA from controls was extracted from peripheral blood specimens using the TIANamp Blood DNA Kit (Tiangen, Beijing, China). DNA purity and concentration were measured using a UV absorption spectrophotometer (Nano Drop Technologies Inc.). Genotyping analysis was conducted using TaqMan PCR master mix and ABI Prism 7900HT genetic detection system through real-time PCR [39]. A random selection of 5% samples was used as positive and negative controls to ensure the accuracy of genotyping results.

### Statistical analyses

We conducted a χ^2^ test to evaluate the heterogeneity of genotypes and ages between patients and controls. We assessed the association between SNP and ovarian cancer risk using a generalized linear regression model and calculated crude and adjusted odds ratios (ORs) and 95% confidence intervals (CIs). A χ^2^ chi-square test was performed to evaluate deviation from Hardy–Weinberg equilibrium (HWE) among the control group [40]. All statistical tests were conducted using SAS software (Version 9.4; SAS Institute, Cary, NC, U.S.A.), with a two-sided *P*-value of < 0.05 considered significant.

## Results

### Association of PIWIL1 genes SNPs and EOC risk

**Fig. 1:**
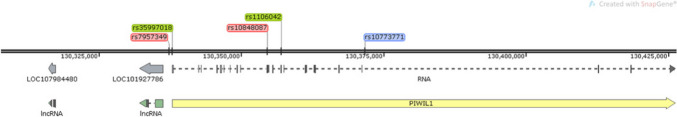
SNPs location of *PIWIL1* gene. The picture shows the SNP sites used in this paper. The polymorphism of the ovarian cancer risk gene are shown in red, and the site that has a protective effect on EOC are shown in blue. Green was not found to be significant

In this study, five *PIWIL1* gene SNPs (rs10848087 G > A, rs35997018 T > C, rs10773771 C > T, rs7957349 G > C, and rs1106042 G > A) were genotyped in 288 EOC samples and 361 age-matched healthy controls. The study found that three SNPs (rs10848087 G > A, rs10773771 C > T, and rs7957349 G > C) were in Hardy–Weinberg equilibrium (*p* > 0.05). However, the rs35997018 and rs1106042 variants were not (*p* < 0.05). Single-locus analysis was conducted to examine the relationship between rs10848087 G > A, rs10773771 C > T, rs7957349 G > C and EOC risk. The results revealed that rs10848087 AA (adjusted OR = 5.654, 95% CI = 1.562–20.464, *p* = 0.0083) and rs7957349 CC (adjusted OR = 2.984, 95% CI = 1.491–5.972, *p* = 0.002) variants significantly increased the susceptibility of EOC. On the other hand, rs10773771 CC (adjusted OR = 0.573, 95% CI = 0.404–0.812, *p* = 0.0018) was associated with a decreased risk of EOC (Table 1). Location of *PIWIL1* gene polymorphisms that may influence cancer risk (Fig. 1).

**Table 1:** Logistic regression analysis of associations between *PIWIL1* polymorphisms and EOC susceptibility

Genotype	Cases	Controls	P^a^	Crude OR	P	Adjusted OR	P^b^
	(*N* = 288)	(*N* = 391)		(95% CI)		(95% CI) b	
rs10848087 G > A (HWE = 0.810)							
GG	202 (79.84)	284 (81.14)		1.00		1.00	
GA	39 (15.42)	63 (18.00)		0.849 (0.551–1.309)	0.4584	0.830 (0.536–1.285)	0.4022
AA	12 (4.74)	3 (0.86)		5.485 (1.531–19.653)	0.0089	5.368 (1.486–19.385)	0.0103
Additive			0.0085	1.261 (0.897–1.775)	0.1823	1.232 (0.872–1.741)	0.2370
Dominant	51 (20.16)	66 (18.86)	0.6901	1.087 (0.723–1.633)	0.6891	1.054 (0.697–1.593)	0.8042
Recessive	241 (95.26)	347 (99.14)	0.0025	5.759 (1.608–20.627)	0.0072	5.654 (1.562–20.464)	0.0083
rs35997018 T > C (HWE = 0.040)							
TT	129 (54.43)	170 (48.57)		1.00		1.00	
TC	102 (43.04)	159 (45.43)		0.752 (0.547–1.034)	0.0793	0.773 (0.561–1.066)	0.1170
CC	6 (2.53)	21 (6.00)		0.335 (0.132–0.848)	0.02	0.351 (0.138–0.895)	0.0283
Additive			0.0894	0.758 (0.569–1.010)	0.0581	0.780 (0.583–1.044)	0.0946
Dominant	108 (45.57)	180 (51.43)	0.1636	0.791 (0.568–1.100)	0.1638	0.818 (0.585–1.143)	0.2396
Recessive	231 (97.47)	329 (94.00)	0.0490	0.407 (0.162–1.024)	0.0562	0.423 (0.167–1.074)	0.0703
rs10773771 C > T (HWE = 0.391)							
CC	106 (50.00)	130 (36.83)		1.00		1.00	
CT	59 (27.83)	162 (45.89)		0.336 (0.234–0.484)	< 0.0001	0.335 (0.232–0.485)	< 0.0001
TT	47 (22.17)	61 (17.28)		0.711 (0.461–1.098)	0.1241	0.714 (0.460–1.107)	0.1317
Additive			0.0001	0.861 (0.684–1.084)	0.2021	0.854 (0.677–1.077)	0.1819
Dominant	106 (50.00)	223 (63.17)	0.0021	0.583 (0.413–0.823)	0.0022	0.573 (0.404–0.812)	0.0018
Recessive	165 (77.83)	292 (82.72)	0.1524	1.364 (0.891–2.087)	0.1533	1.358 (0.884–2.088)	0.1625
rs7957349 G > C (HWE = 0.289)							
GG	164 (65.60)	214 (60.62)		1.00		1.00	
GC	60 (24.00)	126 (35.69)		0.594 (0.415–0.851)	0.0045	0.612 (0.426–0.878)	0.0077
CC	26 (10.40)	13 (3.68)		2.495 (1.250–4.979)	0.0095	2.487 (1.239–4.990)	0.0104
Additive			0.0002	1.047 (0.805–1.364)	0.7305	1.056 (0.809–1.379)	0.6892
Dominant	86 (34.40)	139 (39.38)	0.2132	0.807 (0.576–1.131)	0.2135	0.822 (0.584–1.156)	0.2594
Recessive	224 (89.60)	340 (96.32)	0.0010	3.036 (1.528–6.033)	0.0015	2.984 (1.491–5.972)	0.0020
rs1106042 G > A (HWE = 0.031)							
GG	216 (76.87)	259 (73.79)		1.00		1.00	
GA	55 (19.57)	79 (22.51)		0.934 (0.635–1.373)	0.7272	0.949 (0.643–1.400)	0.7919
AA	10 (3.56)	13 (3.70)		1.032 (0.444–2.395)	0.9424	1.005 (0.429–2.353)	0.9907
Additive			0.6572	0.889 (0.658–1.201)	0.4440	0.886 (0.654–1.201)	0.4358
Dominant	65 (23.13)	92 (26.21)	0.3733	0.847 (0.588–1.221)	0.3736	0.849 (0.587–1.229)	0.3857
Recessive	271 (96.44)	338 (96.30)	0.9299	0.959 (0.414–2.222)	0.9231	0.922 (0.394–2.157)	0.8508

*Abbreviations*: *EOC* knee osteoarthritis, *HWE* Hardy–Weinberg equilibrium, *OR* odds ratios, *CI* confidence interval

^a^χ2 test for genotype distributions between KOA cases and cancer-free controls

^b^Adjusted for age and gender

### Stratification analysis of rs10848087, rs7957349 and rs10773771 with EOC susceptibility

Genotype rs10848087 AA had a harmful effect on cases with metastasis (adjusted OR = 8.919, 95% CI = 2.141–37.165, *p* = 0.0027), clinical stage 1 (adjusted OR = 7.301, 95% CI = 1.348–39.537, *p* = 0.0211), clinical stage 3 (adjusted OR = 6.490,95% CI = 1.403–30.022, *p* = 0.0167), low pathological grade (adjusted OR = 5.908, 95% CI = 1.143–30.546, *p* = 0.0341), high pathological grade (adjusted OR = 6.718, 95% CI = 1.770–25.493, *p* = 0.0051), single tumor (adjusted OR = 5.443, 95% CI = 1.016–29.161, *p* = 0.0479), multiple tumors (adjusted OR = 4.602, 95% CI = 1.008–21.012, *p* = 0.0488), tumor size > 3 cm (adjusted OR = 6.503, 95% CI = 1.381–30.615, *p* = 0.0178), tumor size ≤ 3 cm (adjusted OR = 5.523, 95% CI = 1.392–21.912, *p* = 0.0151), pregnant times ≤ 3 (adjusted OR = 7.386, 95% CI = 0.650–29.486, *p* = 0.0046), pregnant times > 3 (adjusted OR = 4.725, 95% CI = 1.096–20.367, *p* = 0.0373), pre-menopause (adjusted OR = 6.172, 95% CI = 1.437–26.516, *p* = 0.0144), strong positive ER expression (adjusted OR = 6.232, 95% CI = 1.202–32.300, *p* = 0.0293), strong positive PR expression (adjusted OR = 6.584, 95% CI = 1.048–41.362, *p* = 0.0444), strong positive PAX8 expression (adjusted OR = 5.885, 95% CI = 1.164–30.005, *p* = 0.0330), negative/mild positive wild p53 expression (adjusted OR = 6.172, 95% CI = 1.773–37.671, *p* = 0.0071), strong positive wild p53 expression (adjusted OR = 4.717, 95% CI = 1.210–18.395, *p* = 0.0255), mutant p53 expression (adjusted OR = 5.723, 95% CI = 1.326–24.700, *p* = 0.0194) no mutant p53 expression (adjusted OR = 5.969, 95% CI = 1.496–23.817, *p* = 0.0114), strong positive WT1 expression (adjusted OR = 10.408, 95% CI = 2.511–43.135, *p* = 0.0012), strong positive P16 expression (adjusted OR = 12.751, 95% CI = 3.190–50.966, *p* < 0.001), strong positive Ki67 expression (adjusted OR = 10.378, 95% CI = 1.947–55.310, *p* = 0.0061).

The rs7957349 CC was also identified to increase the EOC risk in woman with age ≤ 53 years (adjusted OR = 3.399, 95% CI = 1.387–8.326, *p* = 0.0074), metastasis (adjusted OR = 3.622, 95% CI = 1.542–8.511, *p* = 0.0031), no metastasis (adjusted OR = 2.752, 95% CI = 1.250–6.058, *p* = 0.0119), clinical stage 1 (adjusted OR = 5.870, 95% CI = 2.428–14.191, *p* < 0.0001), low pathological grade (adjusted OR = 3.033, 95% CI = 1.200–7.664, *p* = 0.0190), single tumor (adjusted OR = 4.063, 95% CI = 1.719–9.634, *p* = 0.0014), multiple tumors (adjusted OR = 2.490, 95% CI = 1.028–6.029, *p* = 0.0432), tumor size > 3 cm (adjusted OR = 3.019, 95% CI = 1.137–8.018, *p* = 0.0266), tumor size ≤ 3 cm (adjusted OR = 3.024, 95% CI = 1.421–6.434, *p* = 0.0041), pregnant times ≤ 3 (adjusted OR = 3.768, 95% CI = 1.697–8.368, *p* = 0.0011), pregnant times > 3 (adjusted OR = 2.359, 95% CI = 1.039–5.354, *p* = 0.0402), post-menopause (adjusted OR = 3.020, 95% CI = 1.394–6.544, *p* = 0.0051), pre-menopause (adjusted OR = 3.163, 95% CI = 1.106–9.048, *p* = 0.0317), strong positive wild p53 expression (adjusted OR = 3.460, 95% CI = 1.693–7.072, *p* = 0.0007), no mutant p53 expression (adjusted OR = 4.254, 95% CI = 20.19–8.963, *p* = 0.0001), strong positive Ki67 expression (adjusted OR = 3.126, 95% CI = 1.286–7.602, *p* = 0.0119).

The rs10773771 CT/TT could protect the woman from the risk of EOC on age > 53 years (adjusted OR = 0.390, 95% CI = 0.221–0.688, *p* = 0.0012), metastasis (adjusted OR = 0.529, 95% CI = 0.322–0.870, *p* = 0.0121), no metastasis (adjusted OR = 0.624, 95% CI = 0.411–0.947, *p* = 0.0267), clinical stage 3 (adjusted OR = 0.433, 95% CI = 0.264–0.709, *p* < 0.001), high pathological grade (adjusted OR = 0.563, 95% CI = 0.378–0.839, *p* = 0.0048), multiple tumors (adjusted OR = 0.520, 95% CI = 0.332–0.815, *p* = 0.0043), tumor size ≤ 3 cm (adjusted OR = 0.503, 95% CI = 0.337–0.751, *p* = 0.0008), pregnant times ≤ 3 (adjusted OR = 0.609, 95% CI = 0.383–0.969, *p* = 0.0363), pregnant times > 3 (adjusted OR = 0.532, 95% CI = 0.348–0.813, *p* = 0.0036), post-menopause (adjusted OR = 0.485, 95% CI = 0.323–0.728, *p* < 0.001), strong positive ER expression (adjusted OR = 0.355, 95% CI = 0.202–0.625, *p* < 0.001), negative/mild positive PR expression (adjusted OR = 0.466, 95% CI = 0.222–0.981, *p* = 0.0443), strong positive PR expression (adjusted OR = 0.428, 95% CI = 0.211–0.868, *p* = 0.0187), negative/mild positive PAX8 expression (adjusted OR = 0.245, 95% CI = 0.103–0.583, *p* = 0.0015), strong positive PAX8 expression (adjusted OR = 0.427, 95% CI = 0.244–0.748, *p* = 0.0029), negative/mild wild p53 expression (adjusted OR = 0.502, 95% CI = 0.291–0.868, *p* = 0.0136), strong positive wild p53 expression (adjusted OR = 0.603, 95% CI = 0.407–0.893, *p* = 0.0116), mutant p53 expression (adjusted OR = 0.450, 95% CI = 0.287–0.706, *p* = 0.0005), negative/mild positive WT1 expression (adjusted OR = 0.240, 95% CI = 0.105–0.549, *p* < 0.001), strong positive WT1 expression (adjusted OR = 0.491, 95% CI = 0.293–0.825, *p* = 0.0072), negative/mild positive P16 expression (adjusted OR = 0.294, 95% CI = 0.135–0.641, *p* = 0.0021), strong positive P16 expression (adjusted OR = 0.530, 95% CI = 0.305–0.922, *p* = 0.0247), strong positive Ki67 expression (adjusted OR = 0.445, 95% CI = 0.269–0.736, *p* = 0.0016) (Table 2).

**Table 2:** Stratification analysis of *PIWIL1* polymorphisms with EOC susceptibility

Variables	rs10848087 G > A		Adjusted OR^a^	*P*^a^	rs7957349 G > C		Adjusted OR^a^	*P*^a^	rs10773771 C > T		Adjusted OR^a^	*P*^a^
	(cases/controls)		(95% CI)		(cases/controls)		(95% CI)		(cases/controls)		(95% CI)	
	GG/GA	AA			GG/GC	CC			CC	CT/TT		
Age, years												
> 53	112/347	7/3	3.060 (0.720–13.010)	0.1298	103/340	12/13	2.445 (0.832–7.186)	0.1040	51/130	45/223	0.390 (0.221–0.688)	0.0012
≤ 53	129/347	5/3	> 999.999(< 0.001, > 999.999)	0.9754	121/340	14/13	3.399 (1.387–8.326)	0.0074	55/130	61/223	0.729 (0.467–1.139)	0.1656
Metastasis												
Yes	84/347	6/3	8.919 (2.141–37.165)	0.0027	78/340	11/13	3.622 (1.542–8.511)	0.0031	41/130	39/223	0.529 (0.322–0.870)	0.0121
No	140/347	5/3	4.232 (0.981–18.253)	0.0530	131/340	14/13	2.752 (1.250–6.058)	0.0119	59/130	65/223	0.624 (0.411–0.947)	0.0267
Clinical stage												
1	55/347	3/3	7.301 (1.348–39.537)	0.0211	47/340	11/13	5.870 (2.428–14.191)	< 0.0001	18/130	27/223	0.814 (0.427–1.552)	0.5329
2	41/347	2/3	5.983 (0.962–37.200)	0.0550	41/340	4/13	2.452 (0.760–7.919)	0.1336	17/130	22/223	0.736 (0.376–1.441)	0.3715
3	84/347	4/3	6.490 (1.403–30.022)	0.0167	79/340	5/13	1.680 (0.576–4.903)	0.3424	46/130	36/223	0.433 (0.264–0.709)	0.0009
4	24/347	1/3	4.902 (0.490–49.086)	0.1763	21/340	2/13	2.480 (0.524–11.746)	0.2522	10/130	12/223	0.684 (0.286–1.634)	0.3923
Pathological grade												
low	70/347	3/3	5.908 (1.143–30.546)	0.0341	65/340	8/13	3.033 (1.200–7.664)	0.0190	23/130	28/223	0.672 (0.369–1.224)	0.1942
high	148/347	9/3	6.718 (1.770–25.493)	0.0051	143/340	11/13	1.976 (0.852–4.581)	0.1125	71/130	71/223	0.563 (0.378–0.839)	0.0048
Tumor number												
single	73/347	3/3	5.443 (1.016–29.161)	0.0479	70/340	11/13	4.063 (1.719–9.634)	0.0014	28/130	30/223	0.586 (0.332–1.034)	0.0652
multiple	108/347	4/3	4.602 (1.008–21.012)	0.0488	94/340	9/13	2.490 (1.028–6.029)	0.0432	53/130	49/223	0.520 (0.332–0.815)	0.0043
Tumor size												
> 3 cm	63/347	4/3	6.503 (1.381–30.615)	0.0178	57/340	7/13	3.019 (1.137–8.018)	0.0266	26/130	24/223	0.523 (0.287–0.953)	0.0343
≤ 3 cm	158/347	7/3	5.523 (1.392–21.912)	0.0151	146/340	17/13	3.024 (1.421–6.434)	0.0041	74/130	67/223	0.503 (0.337–0.751)	0.0008
Pregnant times												
≤ 3	104/347	7/3	7.386 (0.650–29.486)	0.0046	96/340	14/13	3.768 (1.697–8.368)	0.0011	45/130	49/223	0.609 (0.383–0.969)	0.0363
> 3	137/347	5/3	4.725 (1.096–20.367)	0.0373	128/340	12/13	2.359 (1.039–5.354)	0.0402	61/130	67/223	0.532 (0.348–0.813)	0.0036
Pausimenia												
post-menopause	177/347	7/3	3.601 (0.861–15.066)	0.0793	159/340	19/13	3.020 (1.394–6.544)	0.0051	80/130	72/223	0.485 (0.323–0.728)	0.0005
pre-menopause	64/347	5/3	6.172 (1.437–26.516)	0.0144	65/340	7/13	3.163 (1.106–9.048)	0.0317	26/130	34/223	0.860 (0.485–1.523)	0.6048
ER expression												
negtive/mild positive	34/347	1/3	.0792 (0.370–38.864)	0.2617	32/340	3/13	2.262 (0.602–8.507)	0.2269	14/130	12/223	0.455 (0.202–1.025)	0.0575
strong positive	67/347	3/3	6.232 (1.202–32.300)	0.0293	63/340	4/13	1.546 (0.484–4.936)	0.4622	37/130	24/223	0.355 (0.202–0.625)	0.0003
PR expression												
negtive/mild positive	34/347	1/3	3.526 (0.354–35.080)	0.2823	32/340	3/13	2.418 (0.653–8.948)	0.1860	17/130	14/223	0.466 (0.222–0.981)	0.0443
strong positive	38/347	2/3	6.584 (1.048–41.362)	0.0444	37/340	4/13	2.771 (0.857–8.961)	0.0887	20/130	15/223	0.428 (0.211–0.868)	0.0187
PAX8 expression												
negtive/mild positive	28/347	1/3	4.394 (0.435–44.426)	0.2099	27/340	1/13	0.945 (0.118–7.555)	0.9572	18/130	8/223	0.245 (0.103–0.583)	0.0015
strong positive	62/347	3/3	5.885 (1.164–30.005)	0.0330	62/340	2/13	0.840 (0.185–3.822)	0.8219	34/130	26/223	0.427 (0.244–0.748)	0.0029
Wild p53 expression												
negtive/mild positive	59/347	4/3	6.172 (1.773–37.671)	0.0071	57/340	3/13	1.383 (0.381–5.017)	0.6217	33/130	29/223	0.502 (0.291–0.868)	0.0136
strong positive	182/347	8/3	4.717 (1.210–18.395)	0.0255	167/340	23/13	3.460 (1.693–7.072)	0.0007	73/130	77/223	0.603 (0.407–0.893)	0.0116
Mutant p53 expression												
Yes	112/347	5/3	5.723 (1.326–24.700)	0.0194	110/340	7/13	1.624 (0.626–4.211)	0.3190	57/130	46/223	0.450 (0.287–0.706)	0.0005
No	129/347	7/3	5.969 (1.496–23.817)	0.0114	114/340	19/13	4.254 (20.19–8.963)	0.0001	49/130	60/223	0.700 (0.451–1.085)	0.1108
WT1 expression												
negtive/mild positive	34/347	1/3	3.712 (0.366–37.673)	0.2674	33/340	3/13	2.320 (0.622–8.652)	0.2102	20/130	9/223	0.240 (0.105–0.549)	0.0007
strong positive	74/347	6/3	10.408 (2.511–43.135)	0.0012	75/340	4/13	1.426 (0.450–4.518)	0.5467	38/130	34/223	0.491 (0.293–0.825)	0.0072
P16 expression												
negtive/mild positive	36/347	1/3	3.506 (0.346–35.542)	0.2885	34/340	3/13	2.251 (0.603–8.402)	0.2272	20/130	11/223	0.294 (0.135–0.641)	0.0021
strong positive	66/347	7/3	12.751 (3.190–50.966)	0.0003	64/340	6/13	2.424 (0.884–6.644)	0.0854	31/130	29/223	0.530 (0.305–0.922)	0.0247
ki67 expression												
negtive/mild positive	36/347	3/3	10.378 (1.947–55.310)	0.0061	34/340	3/13	2.252 (0.606–8.375)	0.2257	11/130	13/223	0.540 (0.276–1.484)	0.2985
strong positive	81/347	3/3	4.598 (0.905–23.371)	0.0659	76/340	9/13	3.126 (1.286–7.602)	0.0119	43/130	34/223	0.445 (0.269–0.736)	0.0016

^a^Adjusted for age and gender

### Haplotype analysis of *PIWIL1* gene SNPs correlated with EOC susceptibility

Our study investigated the association between haplotypes of the *PIWIL1* gene SNPs and the risk of EOC. The haplotype containing the wild-type alleles (GCG) was considered the reference group. The haplotype GTG (adjusted OR = 0.688, 95% CI = 0.509–0.926, *p* = 0.014) was significantly associated with a decreased risk of EOC (Table 3).

**Table 3:** Associatioin between inferred haplotypes of the *PIWIL1* genes and EOC risk

Haplotypes	Cases (*n* = 394)	Controls (*n* = 698)	Crude OR (95% CI)	P^a^	Adjusted OR (95% CI)	P^b^
	No.%	No.%				
GCG	167 (42.39)	260 (37.25)	1.000		1.000	
GCC	46 (11.68)	90 (12.89)	0.796 (0.531–1.193)	0.269	0.809 (0.537–1.218)	0.309
GTG	111 (28.17)	246 (35.24)	0.702 (0.522–0.945)	0.020	0.688 (0.509–0.926)	0.014
GTC	21 (5.33)	33 (4.73)	0.991 (0.554–1.771)	0.980	1.010 (0.561–1.817)	0.974
ACC	21 (5.33)	28 (4.01)	1.168 (0.642–2.124)	0.612	1.123 (0.613–2.058)	0.707
ATC	1 (0.25)	0	-	0.979	-	0.979
ATG	1 (0.25)	1 (0.14)	1.557 (0.097–25.061)	0.755	1.048 (0.065–16.953)	0.973
ACG	26 (6.60)	40 (5.73)	1.012 (0.595–1.720)	0.965	1.012 (0.592–1.731)	0.966

^a^The haplotypes order was rs1061027,rs10848087, rs35997018, rs10773771, rs7957349, rs1106042

^b^Obtained in logistic regression models with adjustment for age and gender

### Expression Quantitative Trait Loci (eQTL) analyses

**Fig. 2:**
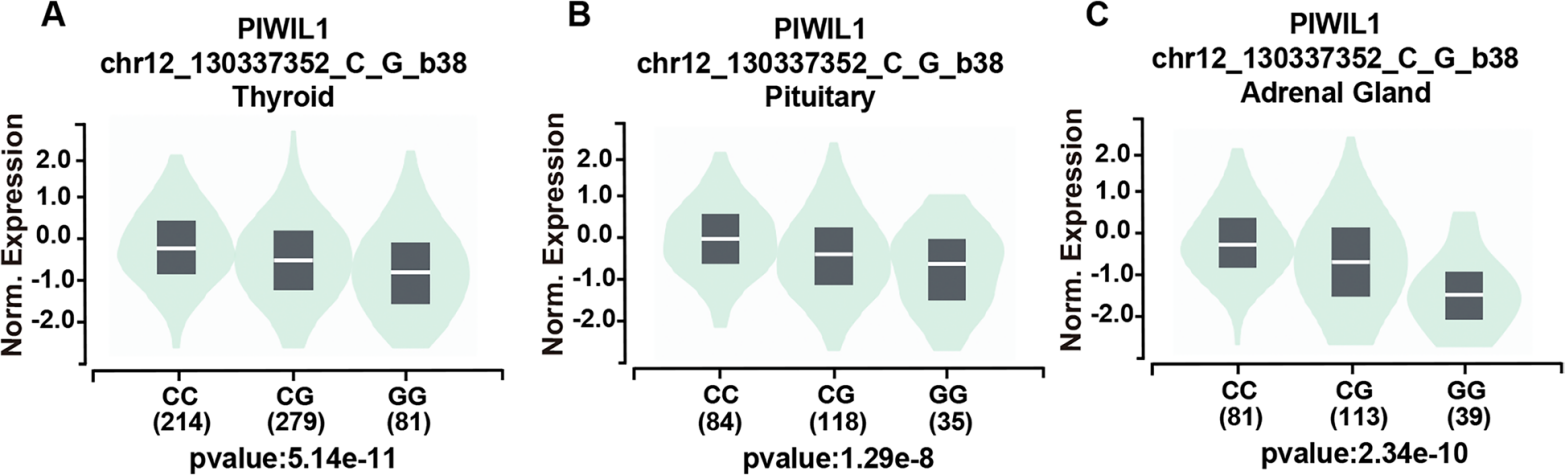
The effect of *PIWIL1* gene polymorphisms on *PIWIL1* expression. *PIWIL1* expression with different genotypes in various organs and tissues was analyzed based on the public database GTEx portal. The expression of *PIWIL1* with different rs7957349 genotypes was shown in the Thyroid (**A**), Pituitary (**B**), and Adrenal (**C**)

To determine the functional relevance of *PIWIL1* rs10848087, rs7957349, and rs10773771, we analyzed released data from GTEx. It revealed that the rs10848087 G > A and rs10773771 C > T were not significantly associated with *PIWIL1* expression. However, the rs7957349 CC genotype had high expression in the thyroid, pituitary, and adrenal glands (Fig. 2).

### SNP-SNP interaction analysis

**Fig. 3:**
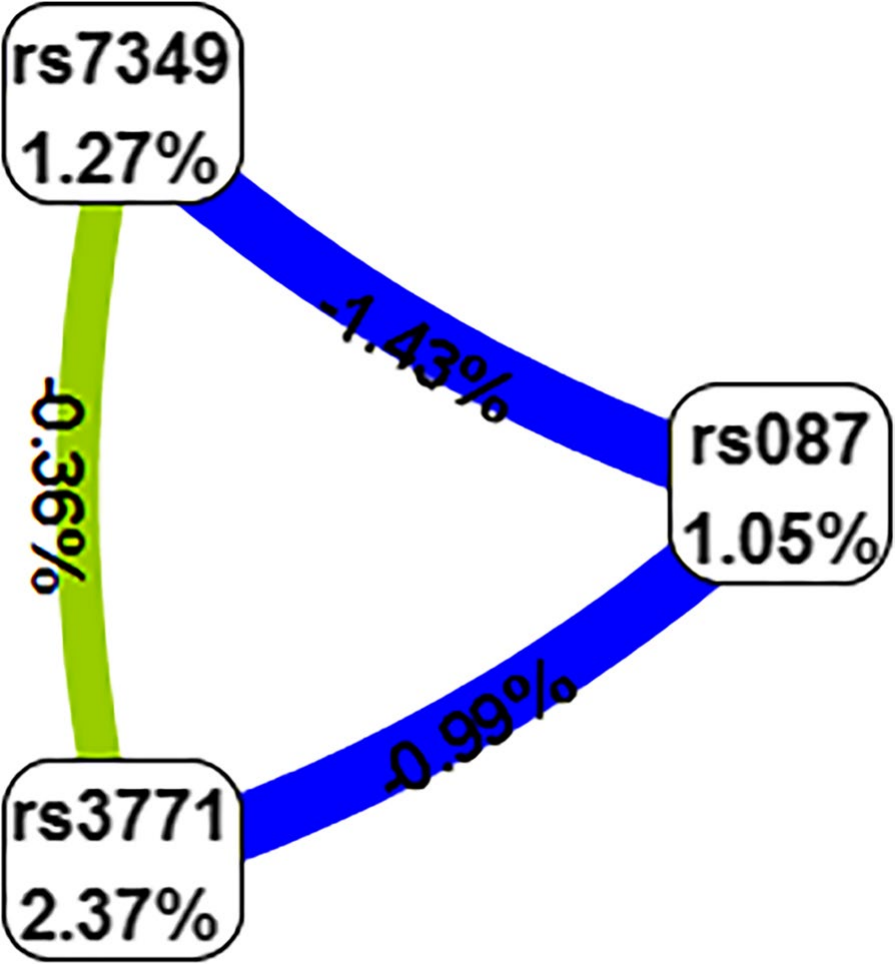
Interaction map for EOC risk. The interaction model describes the percentage of the entropy (information gain) that is explained by each factor or 2-way interaction. Negative entropy (plotted in yellow-green or green) indicates independence or additivity (redundancy)

The MDR analysis revealed that the best statistically significant interaction model predicting a potential EOC risk was that of order three among polymorphisms rs10848087, rs7957349, and rs10773771, *P* = 0.2403 (Table 4). The interaction map shows the following interaction order: *PIWIL1* gene polymorphisms rs7957349 × rs10773771 > rs10773771 × rs10848087 > rs7957349 × rs10848087 with low values of positive entropy or synergism (-0.36%, -0.99%, -1.43%, respectively, shown in green and blue); low entropy values mean redundancy or even independence (Fig. 3).

**Table 4:** Best multifactor dimensionality reduction (MDR) interaction models

Locus number	Testing Accuracy	CVC	OR	95% CI	P
rs10848087, rs7957349 and rs10773771	0.5696	10/10	1.9608	0.6329,6.0745	0.2403

The model was considered as the best model

## Discussion

Our study provides epidemiological evidence supporting the role of piRNA processing gene *PIWIL1* polymorphisms in determining the risk of EOC in southern Chinese women. Through a case–control study, we discovered the potential association between *PIWIL1* gene polymorphisms and the risk of EOC in a population of southern Chinese women. Among the five selected polymorphisms, rs10848087 and rs7957349 were associated with increased EOC risk, while rs10773771 was associated with decreased risk. Our research is to report the association between *PIWIL1* gene polymorphisms and EOC.

Recent reports suggest that population-based polygenic risk scores are strongly associate with breast cancer and EOC risks for *BRCA*1/2 carriers and predict significant differences in absolute risk for women at polygenic risk score distribution extremes [41]*. G*enetic variation in transmembrane transport genes may be linked to an increased risk of EOC across different histologic subtypes. The disruption of cellular transport, including trace elements, hormones, and small molecules, may play a role in the development of EOC [42]. Common SNPS can appear in both gene-coding regions and non-coding regions. Although the probability of occurrence in the coding region is relatively small, they will affect the function of genes and lead to changes in biological traits, which is of great significance in the study of genetic diseases [43]. SNPs alter the binding site of the transcription factors, thereby, affecting the efficiency of transcription and translation of the gene [44]. Additionally, the widespread expression of Piwi-interacting RNA (piRNA) pathway genes in human EOC suggests their involvement in tumorigenesis [45]. The *PIWIL1* gene belongs to the *PIWI* family of miRNA processing cleaving enzymes, is associated with tumor growth, and is a crucial member of the Argonaute protein family. *PIWIL1* can bind with a new class of low scores of non-coding RNA piRNAs, which regulate tumor proliferation, metastasis, invasion, and prognosis [46]. *PIWIL1* is a component of ribonucleoprotein complexes and belongs to the evolutionarily conserved PIWI protein family which plays a crucial role in RNA silencing [47]. Studies have reported that increased expression of the *PIWIL1* gene is associated with various cancers such as endometrial cancer, cervical squamous cell carcinoma, colon cancer, and hepatocellular carcinoma [48, 49]. Recent studies have evaluated the association between *PIWIL1* polymorphisms and the risk of other types of cancers, but no significant associations were observed [50, 51]. Liu et al. found that *PIWIL1* rs10773771 CT/CC variant genotypes were associated with a decreased risk of HCC compared to the wild-type TT. The rs10773771 C allele also enhanced the binding of hsa-miR-1264 to the 3'-UTR of *PIWIL1*. These results suggest that rs10773771 may be linked to HCC by affecting miRNA binding to *PIWIL1* [47]. The SNP rs10773771 is located in the 3'-UTR of *PIWIL1* [52] and can alter the mRNA secondary structure of *PIWIL1*. Moreover, rs10773771 can affect the binding of three miRNAs (hsa-miR-1264, hsa-miR-340, hsa-miR-590-3p) to the 3'-UTR of *PIWIL1*. In a previous study, Liu et al. used reporter gene assays to show that rs10773771 can modify the binding ability of hsa-miR-1264 to the 3'-UTR of *PIWIL1* [47]. In Seo's study, the SNP rs10848087 located in the 5'-UTR of *PIWIL1* was identified as a high-quality SNV associated with Alzheimer's disease susceptibility genes and significant associations with hippocampal volume (Hv) in vivo AD pathologies [53]. On the other hand, research on the upstream transcript variant *PIWIL1* rs7957349 has not been observed recently.

The main strength of our study is its focus on women from South China, which allowed us to gather enough statistical data to identify even minor differences in risk. In this study, we found that the AA genotype of the *PIWIL1* rs10848087 was associated with an increased risk of EOC in pre-menopausal women, as well as in patients with stages I and III tumors and metastasis. Specifically, a reduction of both ER and PR has been detected in metastatic tissue [54]. EOC distant metastases account for approximately 16% of cases, with the most common sites being the pleura (33%), liver (26%), and lung (3%) [55]. The role of hormonal receptor status, specifically ER [56] and PR [57] as prognostic parameters in EOC patients has been extensively investigated. However, the reported results are controversial [58]. Our findings suggested that the AA genotype of *PIWIL1* rs10848087 was associated with a higher risk of EOC in women with strong positive expression of ER and PR. Nuclear transcriptional regulator p53 plays a crucial role in various cellular processes. By binding to DNA, p53 regulates the expression of numerous target genes to maintain homeostasis and genome integrity. In case of DNA damage, p53 can activate DNA repair proteins, halt cell growth at the G1/S transition for DNA repair, and initiate apoptosis if the DNA damage is irreparable [59]. Given its vital function in tumor suppression, it is not surprising that p53 is frequently mutated in cancer, with TP53 mutations present in over 50% of all types of human cancers. Missense mutations in TP53 are particularly common in ovarian cancer (OC), and early-stage cancers have a significantly higher rate of null mutations compared to late-stage disease [60]. In this study, it was found that the CC genotype of the *PIWIL1* rs7957349 variant increased the risk of EOC in women who had strong positive wild p53 expression and no mutant p53 expression. Ki67, encoded by the MKI67 gene, functions as a biological surfactant that disperses mitotic chromosomes. It is commonly used as a proliferation marker in basic research and cancer prognosis [61]. Ki67 is considered a prognostic marker that helps determine the growth fraction of a tumor. Overexpression of Ki67 is associated with malignancy, tumor aggression, poor prognosis, and metastasis [62]. Ki67 is a frequently used tool for evaluating preoperative endocrine in breast cancer [63]. Sehouli's 2019 publication demonstrated that Ki67 is a prognostic factor and a biomarker for predicting therapy outcomes and complete resection in low-grade serous ovarian cancer [64]. However, few studies have evaluated the Ki67 proliferation rate in EOC and the differentiation between histotypes is a frequent problem [65]. Our study revealed that the CC variant of *PIWIL1* rs7957349 was significantly expressed in the Thyroid, Pituitary, and Adrenal glands. Additionally, we observed that this variant might augment the susceptibility of EOC in women with strong positive expression of the Ki67 protein. It may be inferred that the CC variant of the *PIWIL1* rs7957349 gene has an impact on the glands in women, leading to changes in hormone secretion and an increase in the production of Ki67. This variant can be a diagnostic tool for predicting prognosis and assessing survival rates. This study aims to analyze the correlation between Ki67 expression and *PIWIL1* rs7957349 in EOC.

However, limitations of our research include the lack of functional or molecular biology studies to establish the biological significance of the observed associations the small sample size in women, and the presence of rs28416520 was not assessed in the study. The study found that genetic variation in *PIWIL1* genes may increase the risk of EOC, particularly in southern Chinese women. However, there is a weakness in the study due to a small sample size of women, and the contribution of genetic and biological differences to EOC among different ethnic groups is still unclear. Nonetheless, given the observed ovarian cancer health disparities globally and in the U.S., further dedicated studies on this topic are necessary [66]. The underlying mechanisms of the identified associations between rs10848087, rs7957349, rs10773771, and EOC also require further investigation.

Our research has shown that genetic variation in *PIWIL1* is linked to the risk of EOC. Specifically, we have identified an association between three specific genetic markers (rs10848087, rs7957349, and rs10773771) and EOC in southern Chinese women. These markers can potentially serve as biomarkers for EOC susceptibility and aid in determining appropriate chemotherapeutic options. However, further research is needed to understand the underlying mechanisms involved fully.

### Supplementary Information

**Additional file 1: Table S1.** Clinical characteristics of EOC patients and healthy control subjects.

**Additional file 2: Table S2.** DNA quality data.

**Additional file 3: Table R2.** Stratification analysis of PIWIL1 polymorphisms with EOC susceptibility.

## Data Availability

The data that support the findings of this study are available from the corresponding author upon reasonable request.
