# Supplementary material for: Relationship between PIWIL1 gene polymorphisms and epithelial ovarian cancer susceptibility among southern Chinese woman: a three-center case–control study
Source: BMC Cancer. 2023 Nov 27;23:1149. doi: 10.1186/s12885-023-11651-2 (PMC10680212; doi:10.1186/s12885-023-11651-2)
Supplement: Supplementary file 1 — Additional file 1: Table S1. Clinical characteristics of EOC patients and healthy control subjects. [file 12885_2023_11651_MOESM1_ESM.docx]

| **Table S1. Clinical characteristics of EOC patients and healthy control subjects.** | | | |
| --- | --- | --- | --- |
| Varialble | case | control | *P* |
|  | n=288 | n=361 |  |
| Age (years) | N(%) | N(%) | 0.5664 |
| ≤53 | 155(53.82) | 233(64.54) |  |
| ＞53 | 133(46.18) | 128(35.46) |  |
| Pausimenia |  |  |  |
| post-menopause | 206(71.53) | NA |  |
| pre-menopause | 82(28.47) | NA |  |
| Metastasis |  |  |  |
| Yes | 98(34.03) | NA |  |
| No | 171(59.38) | NA |  |
| FIGO stage |  |  |  |
| I | 71(24.65) | NA |  |
| II | 49(17.01) | NA |  |
| III | 93(32.29) | NA |  |
| IV | 25(08.68) | NA |  |
| Pathological grade |  |  |  |
| Low/middle | 85(29.51) | NA |  |
| High | 171(59.38) | NA |  |
| Tumor number |  |  |  |
| Single | 101(35.07) | NA |  |
| Multiple | 117(40.63) | NA |  |
| Tumor size (cm) |  |  |  |
| ≤3 cm | 192(66.67) | NA |  |
| >3 cm | 72(25.00) | NA |  |
| pregnant times |  |  |  |
| ≤3 cm | 126(43.75) | NA |  |
| >3 cm | 162(56.25) | NA |  |
| ER expression |  |  |  |
| negative/mild positive | 38(13.19) | NA |  |
| strong positive | 81(28.13) | NA |  |
| PR expression |  |  |  |
| negative/mild positive | 38(09.72) | NA |  |
| strong positive | 47(16.32) | NA |  |
| PAX8 expression |  |  |  |
| negative/mild positive | 29（10.07） | NA |  |
| strong positive | 72（25.00） | NA |  |
| Wildtype p53 |  |  |  |
| Positive | 66(22.92) | NA |  |
| Negative | 222(77.08) | NA |  |
| Mutant p53 |  |  |  |
| Positive | 133(46.18) | NA |  |
| Negative | 155(53.82) | NA |  |
| WT1 expression |  |  |  |
| negative/mild positive | 37（12.85） | NA |  |
| strong positive | 92（31.94） | NA |  |
| p16 expression |  |  |  |
| negative/mild positive | 38(13.19) | NA |  |
| strong positive | 84(29.17) | NA |  |
| Ki67 expression |  |  |  |
| negative/mild positive | 48(16.67) | NA |  |
| strong positive | 96(33.33) | NA |  |
|  |  |  |  |
|  |  |  |  |
|  |  |  |  |
| EOC, epithelial ovarian cancer; | | |  |
| FIGO, International Federation of Gynaecology and Obstetrics |  |  |  |
| NA = not applicable. |  |  |  |

SD, standard deviation;

^a^ Two-sided χ2 test for distributions between EOC cases and cancer-free controls.
