# Supplementary figures and images for: Relationship between PIWIL1 gene polymorphisms and epithelial ovarian cancer susceptibility among southern Chinese woman: a three-center case–control study
Source: BMC Cancer. 2023 Nov 27;23:1149. doi: 10.1186/s12885-023-11651-2 (PMC10680212; doi:10.1186/s12885-023-11651-2)

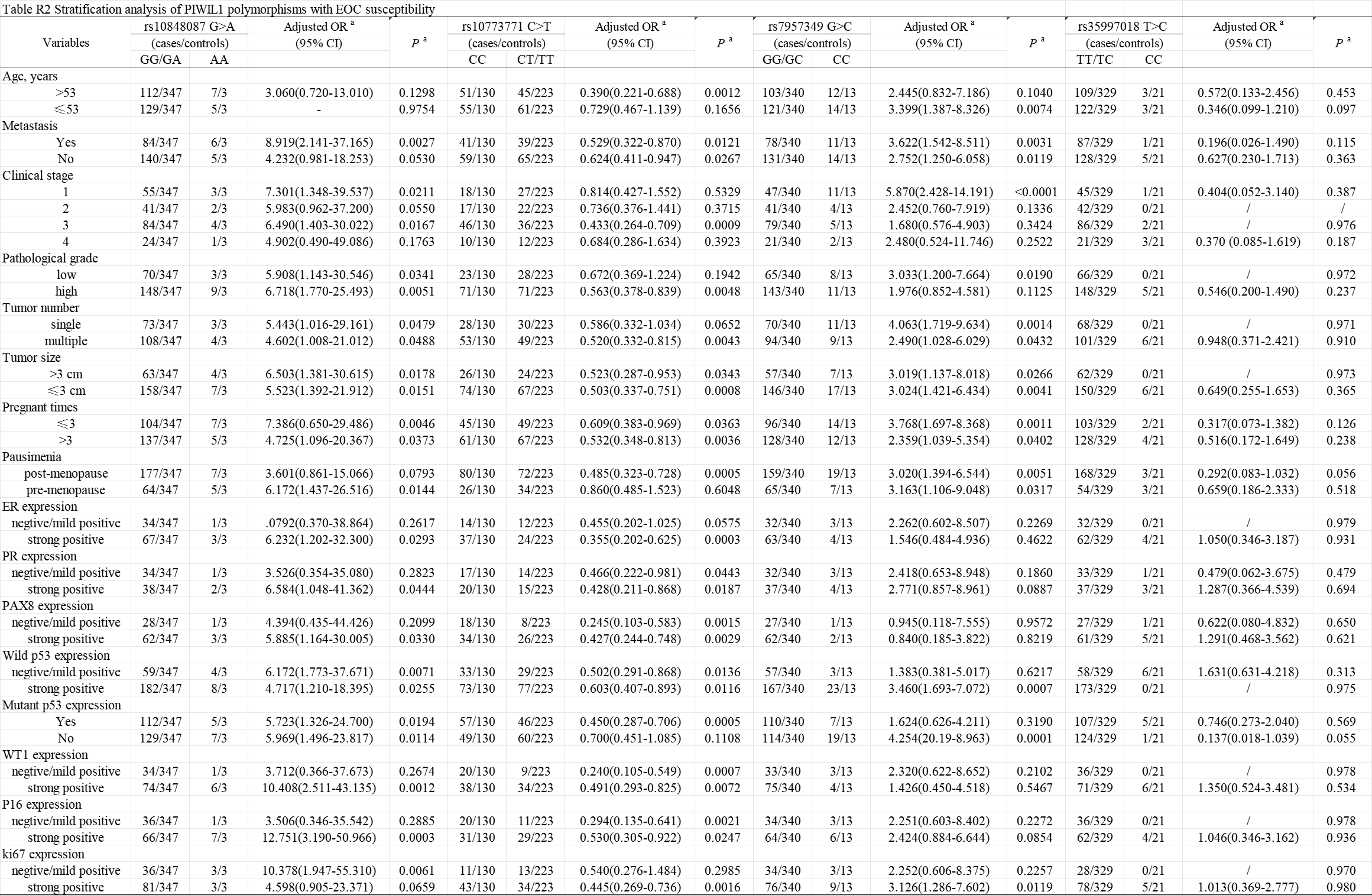

Supplement: Supplementary file 3 — Additional file 3: Table R2. Stratification analysis of PIWIL1 polymorphisms with EOC susceptibility. [file 12885_2023_11651_MOESM3_ESM.docx]
